# All science should inform policy and regulation

**DOI:** 10.1371/journal.pmed.1002576

**Published:** 2018-05-03

**Authors:** John P. A. Ioannidis

**Affiliations:** 1 Stanford Prevention Research Center, Department of Medicine, Department of Health Research and Policy and Department of Biomedical Data Science, Stanford University School of Medicine, Stanford, California, United States of America; 2 Department of Statistics, Stanford University School of Humanities and Science, Stanford, California, United States of America; 3 Meta-Research Innovation Center at Stanford (METRICS), Stanford University, Stanford, California, United States of America

## Abstract

In the context of a recent proposal to exclude research from consideration at the Environmental Protection Agency, John Ioannidis points out that “perceived perfection is not a characteristic of science, but of dogma” and envisions how governments can promote a standard of openness in science.

Not all scientific information is created equal. Large differences exist across topics on how much is known, and with what degree of certainty. Some questions are more difficult to answer, and some research tools are more reliable than others. Not all methods can be applied to answer every question. Credibility depends [1] on how large and rigorous studies are, how well researchers have contained conflicts of interest (financial or other), and how successfully the study design and analysis have limited bias, properly accounting for the complexity inherent in each scientific question. Coordinated efforts among scientists instead of furtive competition help improve the odds of success. Transparency with full sharing of data, protocols and computer codes improves trust in research findings. Re-analysis of data by independent teams adds to that trust and replication in new studies further enhances it.

Scientific findings vary in their credibility. Some findings are beyond reasonable doubt. For example, we have extremely strong evidence that the tobacco pandemic is devastating; that the MMR vaccine is generally safe; that climate change is happening; and that air pollution is a major health hazard. Conversely, our evidence base is notoriously weak on most dietary advice one might hope to give about specific nutrients [2]. Within a given discipline, evidence may be strong for some findings but weak for others. E.g., we have strong evidence for some medical interventions, modest evidence for others, and dismally biased evidence for many.

Our society will benefit from using the best available science for governmental regulation and policy. One can only applaud when governments want to support the best possible science, invest in it, find ways to reduce biases, and provide incentives that bolster transparency, reproducibility, and the application of best methods to address questions that matter. However, perceived perfection is not a characteristic of science, but of dogma. Even the strongest science may have imperfections. In using scientific information for decision-making, it is essential to examine evidence in its totality, recognize its relative strengths and weaknesses, and make the best judgment based on what is available.

Making scientific data, methods, protocols, software, and scripts widely available is an exciting, worthy aspiration [3–5]. Government-based regulatory and funding incentives can be instrumental in making this happen at large scale. However, we should recognize that most of the raw data from past studies are not publicly available. In a random sample of the biomedical literature (2000–2014) [6], none of 268 papers shared all of their raw data. Only one shared a full research protocol. The proportion of studies that have had all their raw data independently re-analyzed is probably less than one in a thousand. The number of studies that have been exactly replicated in new investigations is quite larger, but still a minority in most fields. A new standard currently proposed for the Environmental Protection Agency [7] aims to ban the use of scientific studies for regulatory purposes unless all their raw data are widely available in public and can be reproduced. If the proposed rule is approved, science will be practically eliminated from all decision-making processes. Regulation would then depend uniquely on opinion and whim.

Past collected and analyzed information can and should still be used for decision-making, taking into account any relevant imperfections. While fully transparent and reproducible information should certainly be valued more highly, studies with weaknesses can still offer insights. Some deficiencies may be unavoidable. For example, researchers cannot ethically randomize people to harmful exposures in order to tackle confounding, nor violate informed consent agreements that prohibit open sharing of private data from past studies. Instead of violating ethics, we should focus more on future efforts, informed by what we have learned in the past. When avoidable weaknesses are identified, we can improve rigor, transparency and reproducibility (and, eventually, credibility) for future studies.

Successful examples of rigorous, reproducible research can be used as templates for other fields that are struggling with suboptimal research practices. For example, the pivotal research on the health effects of air pollution is particularly strong. The Six Cities [8] and American Cancer Society [9] studies are exemplary large-scale investigations, with careful application of methods, detailed scrutiny of measurements, replication of findings, and, importantly, detailed re-analysis of results and assessment of their robustness by entirely independent investigators [10]. The re-analysis and sensitivity analyses were conducted by the Health Effects Institute that was funded by stakeholders some of whom may have desired to see opposite conclusions. It would be wonderful, if in the future the same rigorous re-analysis and replication standards could become the standard for all important areas of research that can inform policy.

In the USA and elsewhere, governments are major funders of research and their regulatory mandates provide powerful incentives for best science. Making widely applicable, reproducible research practices and sharing the default option for research (with sparse exceptions, when appropriately justified) will strengthen scientific investigation and maximize its benefits to society at large. Governments can bolster their legacy through such initiatives and scientists would be broadly supportive of such a transformative vision to promote a standard of openness in science.

The opposite scenario, of simply ignoring science that has not yet attained such standards, is a nightmare. On the one hand, we would see governments discarding science at massive scale because of perceived imperfections and impurities. Perhaps worse, we would see scientists respond by becoming politically entrenched dogmatic advocates, falsely believing that they defend science. Even well-intentioned academics, perceiving an attack on science, may be tempted to take an unproductive, hand-waving defensive position: “we have no problem with reproducibility”, “everything is fine”, “science is making progress”. Certainly, science is making progress; with 20 million smart people working in and co-authoring scientific work and with major funding investment, it would be horrible if no progress were made. The issue is how we can accelerate progress. To do this, instead of hiding trash under the carpet, we should make the best use of past work and materialize bigger and better plans for the future. Science is facing a major transformation nowadays, with exponentially more data and far more scientists working on them than ever. Financial and other conflicts are major threats. Many analyses are becoming black boxes and reproducibility problems are widely documented across many fields. Most of the effects pursued by current investigations are of modest size, nowhere close to the huge harms of tobacco or the huge benefits of childhood vaccinations. Many fields lack the high reproducibility standards that are already used in fields such as air pollution and climate change. The scientific enterprise faces great challenges and great opportunities and we need the best research practices in order to succeed [11].

While scientists can work to improve science, governments and regulators can also do better. Most governments around the world have largely neglected the need to support reproducible research practices. Moreover, they have not used science as much as they should. This is particularly worrisome when the evidence is strong, yet governments have not acted forcefully enough. It is a scandal that we continue to allow companies to make money from selling tobacco products, despite expecting about 1 billion tobacco-related deaths in the next 100 years, a Holocaust equivalent of lost lives repeated every year. It is a scandal that the response of governments to climate change and pollution has not been more decisive. It is a scandal that we don’t have higher standards for drugs, biologics, and devices. It is a scandal that people die from measles in the 21^st^ century. Current governments have plenty of room to improve over the mediocre performance of their predecessors. They can do this by using, not discarding, science.
